# Mismatch between registration possibilities and patients’ local health needs, a simulated patient survey in the Paris metropolitan area

**DOI:** 10.1186/s12960-025-01020-4

**Published:** 2025-11-03

**Authors:** Raphaëlle Delpech, Henri Panjo, Alexis Costalat, Frédérique Noël, Laurent Rigal

**Affiliations:** 1 Department of General Practice, Département de Médecine Générale, University of Paris-Saclay, Bureau 326, 63 Rue Gabriel PÉRI, Gyf-sur-Yvette, France; 2 CESP (Centre for Research in Epidemiology and Population Health), Inserm U1018, University of Paris-Saclay, UVSQ, Gender, Sexual and Reproductive Health Team, Paris, France

**Keywords:** Primary care access, Simulated patient, Patient registration, Home visit, Practice management, Primary care

## Abstract

**Objectives:**

We studied the association between GPs’ characteristics and the places they practise, in terms of the supply and demand for primary care and of the registration of new patients for ongoing care at the office or for house calls.

**Study design:**

An exhaustive simulated patient survey enabled us to determine the GPs practising in the Paris metropolitan region who were accepting new patients for registration for continuing care at their office and/or for house calls.

**Methods:**

We studied the associations between the characteristics of GPs who were accepting new patient registrations and those describing their office location.

**Results:**

In 2017–2018, we contacted 8171 physicians (87.6% of the GPs in the region), 49.70% were willing to register a new patient for office visits and 18.7% for house calls. In both situations (office and visit), doctors who most frequently agreed to register new patients were men in solo practices, who had no secretary and did not practise alternative medicine. GPs in areas with low levels of deprivation and relatively few individuals with costly chronic diseases agreed more frequently than those elsewhere to register new patients. No characteristic describing the supply of primary care was associated with agreement to register new patients.

**Conclusions:**

The difficulties of finding a GP in the most deprived areas and with the most people with chronic diseases suggest the need to develop policies facilitating the settlement of new doctors in such areas.

**Supplementary Information:**

The online version contains supplementary material available at 10.1186/s12960-025-01020-4.

## Introduction

In many countries, registration with a general practitioner (GP), that is, a primary care physician, determines the extent of access to the health-care system [1, 2]. GPs thus play the role of gatekeeper, and access is restricted for unregistered patients (most often through a financial penalty), especially to second line (specialist) care [3, 4]. Nonetheless some GPs refuse to agree to register new patients. According to estimates in France, half of all GPs do not register new patients, and 11% of the population is not registered with a GP [5, 6]. Similar findings come from Canada, where 15% of the population is not registered. These individuals report that the primary reason is refusal by GPs [7]. In countries that condition access to the health-care system on registration with a GP, patients who have difficulty in finding a doctor who will register them risk renouncing or at least delaying some care. In France, unregistered patients are reimbursed less than half of what registered patients for doctors’ visits (30% instead of 70%) and does not have access to secondary care, which is conditioned on a prerequisite referral by a GP.

To our knowledge, the characteristics of GPs who agree or refuse to register new patients have never been studied. Nonetheless we know that some GPs, faced with populations who need more care than they feel able to offer, choose the strategy of limiting the size of their patient list [8–10]. We can thus hypothesise that GPs in areas where the mismatch between supply and demand is strongest are most likely to refuse to accept patients. It is also reasonable to suppose that refusals will be more frequent in the areas with the most severe socioeconomic deprivation—which are also generally those where the care supply is lowest (the inverse care law [11–13]).

Doctors’ working time is associated with both the quantity and kind of care they offer [14] and their personal characteristics: gender [15], age [16], and office organisation [17]. Thus these characteristics may also be associated with their decision to agree or refuse to register new patients.

Our objective was to study the association between GPs’ characteristics and the places they practise, in terms of the supply and demand for primary care and of the registration of new patients for ongoing care at the office or for house calls (because GPs perform increasingly fewer such visits, registration for them is more difficult [18]).

We tested these two hypotheses in Ile-de-France, a French region containing a wide variety of characteristics related to its population density [19–21] and socioeconomic context [22] that make it possible for us to study different practice settings. This region is the richest in France (accounting for 30% of the country’s GNP) and the second richest in Europe [23], but it is also the French region with the greatest level of inequality [24]. As elsewhere in France, its inhabitants encounter difficulties in registering with a GP; 12% of its population is not registered [5].

## Methods

### Recruitment of doctors

We collected a list of all GPs practising in Ile-de-France from the national health insurance fund directory, set up to aid the insured to find doctors and accessible free of charge on line (http://annuairesante.ameli.fr/). We were thus able to obtain the addresses and telephone number of all practitioners. We contacted only the GPs providing medical care and follow-up over time, in accordance with the tasks assigned to primary-care physicians [25]. GPs providing only specialised, emergency, or preventive care were excluded.

### Simulated patient survey

The offices of the GPs identified were contacted by telephone between January 2017 and December 2018. Trained investigators using a predefined scenario (Appendix 1) presented themselves as having moved into the neighbourhood recently and looking for a new local physician to be registered with, to provide office care. If the answer to this question was positive, they then asked if registration for house-call follow-up was possible for an elderly relative. As in real-life conditions, these questions could be answered either by the office secretary or directly by the doctor. When the response to the first request was negative, house-call visit registration was not mentioned and was considered impossible. If the telephone call was not answered the first time, the investigator called back up to three times, on different days and at different hours. Doctors whose offices could not be reached were excluded. Those whose gender was neither mentioned in the directory nor accessible during the telephone interviews were excluded from the analyses considering gender.

### Outcomes

Our primary outcome measure was the ability of the simulated patient to be registered with the GP for office care, and the secondary outcome registration for at-home care.

### Explanatory variables

We had two types of explanatory variables: those describing the GPs’ characteristics and those describing their office location in terms of the supply and demand for primary care. Their characteristics were collected online from the directory. The information collected comprised gender, the practice of alternative medicine, such as homeopathy or acupuncture, a solo or group practice, and the billing of fees exceeding the standard reimbursement for services. One variable was collected during the telephone interview: whether the doctor had a secretary.

The variables describing the characteristics of their office came from the last census (2016) available online. They were collected at two nested geographic levels: the local neighbourhood (defined by the census block) and the municipality. These characteristics were divided into two categories [26]:

- Characteristics describing the demand for primary care: density of inhabitants; proportion of people in different age groups (≤ 6 years, ≥ 75 years); proportion of the population that had moved during the year; proportion of the population in each tertile of the social deprivation index (SDI); proportion of the population reporting a costly chronic disease to the health insurance fund (long-term disease, ALD). All of these characteristics were collected at the scale of the neighbourhood, except for the proportion of the population with an ALD, which was assessed at the scale of the municipality.
- Characteristics describing the supply of primary care—the number of GP consultations accessible per inhabitant and per year, estimated by a gravity model (the Local Potential Accessibility (LPA) in France) [27–30]—and the distance in minutes to the nearest emergency department were both assessed at the municipal level.

### Statistical analyses

The associations between the outcomes and all of the explanatory variables were analysed with a logistic mixed model with random intercepts and three levels (GP, neighbourhoods, and municipality). Accordingly, two multivariate model were constructed; the first described registration for office care and the second registration for house calls. We used SAS 9.4 software.

The study was reported to the National Data Protection Authority (*commission nationale de l’informatique et des libertés*), which is responsible for ethical issues in research and the protection of individuals from illegal or inappropriate electronic data collection. All doctors were subsequently notified that they had participated and could request to be excluded. None asked to be excluded.

This manuscript was prepared in accordance with the STROBE (Strengthening the Reporting of Observational Studies in Epidemiology) guidelines for reporting observational studies.

## Results

**Fig. 1:**
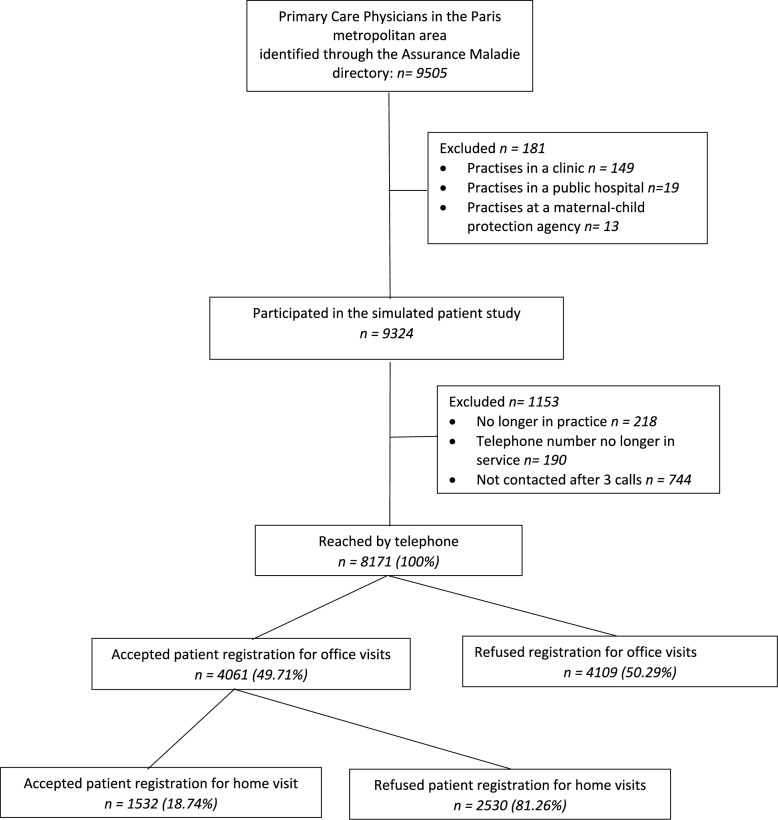
Survey flow chart

Among the 9505 PCPs identified in the directory, 1334 (14.0%) were excluded, mainly because they were unreachable (Fig. 1). Of the 8171 PCPs contacted, 4062 (49.70%) were willing to register a new patient for office visits and 1532 (18.7%) willing to register a new patient for house calls; 56 doctors were excluded from the analyses, because their gender was unknown (Table 1).

**Table 1:** Characteristics of general practitioners and of their offices (N = 8115)

		N (%)
**GPs**		
Gender	Men	5036 (62.06)
	Women	3079 (37.94)
Sector	Sector I	6246 (76.97)
	Sector II	1869 (23.03)
Practice of alternative medicine	No	7201 (88.74)
	Yes	914 (11.26)
Group	Solo	4341 (53.49)
	Group	3774 (46.51)
Secretary	Yes	5784 (71.28)
	No	2331 (28.72)
**PRACTICE NEIGHBOURHOOD**		
**Demographic characteristics**^a^		
Population density (Inhab/km^2^)	Mean (standard deviation	16,337 (15,369)
	T1 = [0.00–3400]	1616 (19.85)
	T2 = [3401–12849]	3039 (37.48)
	T3 = [12958–133932]	3460 (42.67)
Proportion of the population aged 6 years or younger (%)	Mean (standard deviation	7.70 (2.31)
	T1 = [0.42–6.58]	2719 (33.51)
	T2 = [6.58–8.54]	2781 (34.27)
	T3 = [8.55–19.58]	2615 (32.22)
Proportion of the population aged 75 years or older (%)	Mean (standard deviation	7.48 (3.55)
	T1 = [0.00–5.09]	2041 (25.13)
	T2 = [5.10–7.76]	2667 (32.86)
	T3 = [7.77–28.91]	3407 (42.00)
Proportion of the population that moved during the year (%)	Mean (standard deviation	12.12 (2.480)
	T1 = [5.93–10.95]	599 (7.38)
	T2 = [10.96–13.22]	2206 (27.19)
	T3 = [13.22–46.78]	5310 (65.43)
**Socioeconomic characteristics**^a^		
Social deprivation index	T1	2682 (33.05)
	T2	2713 (33.46)
	T3	2720 (33.52)
**Care supply**^b^		
N consultations available per inhabitant per year (2018)	Mean (standard deviation)	3.44 (0.908)
	T1 = [1.01–2.37]	800 (9.85)
	T2 = [3.38–3.02]	2130 (26.25)
	T3 = [3.03–5.95]	5185 (36.90)
Distance to nearest emergency care unit (min)	Mean (standard deviation)	11.81 (8.108)
	T1 = [0–13.0]	4097 (50.48)
	T2 = [14.0–18.0]	2549 (31.41)
	T3 = [19.0–30.0]	1469 (18.10)
Proportion of general practitioners aged 65 years or older (%)	Mean (standard deviation)	38.30 (15.11)
	T1 = [0–33.30]	2701 (33.29)
	T2 = [34.10–44.80]	2735 (33.71)
	T3 = [45.00–100.0]	2679 (33.00)
**Population health**^b^		
N GP consultations used per inhabitant and per municipality	Mean (standard deviation)	3.776 (0.334)
	T1 = [2.87–3.57]	1934 (23.82)
	T2 = [3.57–3.80]	2308 (28.44)
	T3 = [3.81–4.73]	3873 (47.73)
Proportion of the population reporting an ALD (chronic costly disease)(%)	Mean (standard deviation)	17.62 (2.227)
	T1 = [8.10–14.7]	2474 (30.48)
	T2 = [14.8–16.3]	3309 (40.78)
	T3 = [16.5–27.6]	2332 (28.74)

^a^Tertiles of distribution in Ile-de-France by census block

^B^Tertiles of distribution in Ile-de-France by municipality

### GP characteristics associated with registration (Tables 2 and 3)

**Table 2:** Characteristics of GPs, their neighbourhood, and registration of new patients for office visits, multivariate analysis (N = 8115)

			OR [95% CI]^c^	*P* value
**GPs**				
Gender	Men	50.87	**1.29 [1.04–1.37]**	**0.0002**
	Women	49.13	1	
Sector	Sector I	51.06	**1.20 [1.04–1.38]**	**0.0120**
	Sector II	48.94	1	
Group	Solo	50.82	**1.32 [1.17–1.48]**	** < 10** ^**–4**^
	Group	42.27	1	
Secretary	No	55.07	**1.59 [1.40–1.79]**	** < 10** ^**–4**^
	Yes	44.93	**1**	
Practice of alternative medicine	No	52.35	**3.23 [2.70–3.87]**	** < 10** ^**–4**^
	Yes	27.13	**1**	
**PRACTICE NEIGHBOURHOOD**				
**Characteristics describing the demand for primary care**				
Population density (Inhab/km2)a	T1 (least dense)	44.22	1	**0.0136**
	T2	45.25	1.02 [0.84–1.24]	
	T3 (most dense)	55.72	**1.31 [1.05–1.64]**	
Proportion of the population aged 6 years or younger (%)	T1 (lowest)	49.35	0.98 [0.82–1.17]	0.7827
	T2	49.23	0.93 [0.75–1.15]	
	T3 (highest)	50.21	1	
Proportion of the population aged 75 years or older (%)	T1 (lowest)	51.15	1.07 [0.91–1.39]	0.4128
	T2	51.22	1.14 [0.94–1.39]	
	T3 (highest)	47.37	1	
Proportion of the population that moved during the year	T1 (lowest)	49.49	**1.60 [1.15–2.23]**	**0.0215**
	T2	51.37	1.09 [0.88–1.34]	
	T3 (highest)	47.65	1	
Proportion of the population reporting an ALD (costly chronic disease)(%)^b^	T1 (lowest)	62.80	**1.40 [1.06–1.86]**	**0.0316**
	T2	51.97	1.05 [0.83–1.32]	
	T3 (highest)	50.30	1	
Social deprivation index^**a**^	T1 (most disadvantaged)	47.94	1	**0.0015**
	T2	45.99	1.05 [0.79–1.40]	
	T3 (least disadvantaged)	53.09	**1.11 [0.83–1.46]**	
**Characteristics describing the supply of care**				
N consultations available per inhabitant per year (2018)	T1 (lowest)	45.06	1	0.7609
	T2	48.26	1.00 [0.75–1.34]	
	T3 (highest)	50.70	1.03 [0.78–1.36]	
Distance to nearest emergency department (min)	T1 (lowest)	55.81	1	0.7185
	T2	51.05	0.92 [0.74–1.13]	
	T3 (highest)	57.89	0.97 [0.74–1.27]	
Proportion of general practitioners aged 65 years or more (%)	T1 (lowest)	46.57	1	0.3789
	T2	51.88	1.12 [0.88–1.43]	
	T3 (highest)	47.95	1.01 [0.81–1.27]	

Bolded values indicate statistically significant differences (*p* value < 0.05)

^a^Tertiles of distribution in Ile-de-France by census block

^b^Tertiles of distribution in Ile-de-France by municipality

^c^OR: odds ratio; CI: confidence interval

**Table 3:** Characteristics of GPs, their neighbourhood, and registration of new patients for office visits, multivariate analysis (N = 8115)

			OR [95% CI]^c^	*P* value
**GPs**				
Gender	Men	20.79	**1.45 [1.27–1.66]**	** < 10** ^**–4**^
	Women	15.62	1	
Practice sector	Sector II	19.00	1.01 [0.86–1.20]	0.8802
	Sector I	18.25	1	
Group	Solo	16.82	**1.18 [1.03–1.36]**	**0.0193**
	Group	21.14	**1**	
Secretary	No	26.08	**1.70 [1.48–1.96]**	** < 10** ^**–4**^
	Yes	15.90	**1**	
Practice of alternative medicine	No	20.17	**3.07 [2.35–4.00]**	** < 10** ^**–4**^
	Yes	8.21	**1**	
**PRACTICE NEIGHBOURHOOD**				
**Characteristics describing the demand for primary care**				
Population density (Inhab/km2)a	T1 (least dense)	21.18	**1**	**0.0401**
	T2	16.64	**0.75 [0.62–0.97]**	
	T3 (most dense)	19.68	**0.74 [0.58–0.95]**	
Proportion of the population aged 6 years or younger (%)	T1 (lowest)	20.27	0.93 [0.72–1.19]	0.6742
	T2	18.95	1.02 [0.83–1.25]	
	T3 (highest)	17.32	1	
Proportion of the population aged 75 years or older (%)	T1 (lowest)	18.13	1.00 [0.80–1.26]	0.8558
	T2	19.23	1.05 [0.87–1.26]	
	T3 (highest)	19.02	1	
Proportion of the population that moved during the year	T1 (lowest)	19.03	1.39 [0.98–1.98]	0.1195
	T2	18.88	1.19 [0.95–1.49]	
	T3 (highest)	18.57	1	
Proportion of the population reporting an ALD (chronic costly disease)(%)b	T1 (lowest)	26.83	**1.40 [1.03–1.88]**	**0.0091**
	T2	24.02	0.95 [0.74–1.22]	
	T3 (highest)	25.44	1	
Social deprivation index^**a**^	T1 (most disadvantaged)	16.33	1	**0.0371**
	T2	17.63	1.11 [0.87–1.40]	
	T3 (least disadvantaged)	21.35	**1.41 [1.07–1.86]**	
**Characteristics describing the supply of care**				
N consultations available per inhabitant per year (2018)	T1 (lowest)	17.52	1	0.3395
	T2	17.04	0.97 [0.70–1.33]	
	T3 (highest)	19.76	1.14 [0.84–1.54]	
Distance nearest emergency unit (in min)	T1 (lowest)	22.87	1	0.1669
	T2	23.68	1.05 [0.84–1.31]	
	T3 (highest)	33.33	1.28 [0.97–1.70]	
Proportion of general practitioners aged 65 years or more (%)	T1 (lowest)	17.69	1	0.6306
	T2	18.22	1.11 [0.87–1.42]	
	T3 (highest)	19.30	0.99 [0.78–1.26]	

Bolded values indicate statistically significant differences (*p* value < 0.05)

^a^Tertiles of distribution in Ile-de-France by census block

^b^Tertiles of distribution in Ile-de-France by municipality

^c^*OR* odds ratio; *CI* confidence interval

Male doctors not charging fees exceeding the standard reimbursement for services, practising alone and without a secretary or answering service, agreed to register new patients more often—for both office and home visits. GPs who practice alternative medicine were less likely to register new patients for office care but did not differ in their rate of registrations for house calls.

### Characteristics describing the practice neighbourhood associated with registration (Tables 2 and 3)

No characteristics describing the supply of primary care in the neighbourhood were associated with registration for care at the doctor’s office or at home.

Two characteristics describing the supply of primary care in the neighbourhood had similar associations with registration for both office and home care. The lower the level of social deprivation and the lower the ALD rate, the more likely GPs in those neighbourhoods were to agree to register new patients—for home as well as office care (with a gradient).

Another characteristic describing the demand for care in a neighbourhood is its population density. It was also significantly associated with registration for office as well as home care, but in different directions. That is, the denser the area, the lower the rate of registration agreement, and inversely, the higher it was for continued care at home, with a gradient. Finally, the proportion of the population that had moved during the year was associated with successful registration for office care, with a gradient (the lower the proportion of patients who had moved, the higher the rate of new patient registration for office care); and without a gradient for registration for care at home.

## Discussion

### Summary

Fewer than half the GPs agreed to register new patients for office care, and only almost one in five for house calls. In both situations, the doctors who most frequently agreed to register new patients were men, in solo practices, with neither a secretary nor any particular type of practice. GPs in areas with little deprivation and with relatively few individuals with costly chronic diseases agreed more frequently than those elsewhere to register new patients for continuing care at the office and even at home. The population density of the neighbourhood was associated positively with registration for office care and negatively with registration for home care. Finally, no characteristic describing the supply of primary care was associated with agreement to register new patients.

### Comparisons and interpretations

#### GP characteristics associated with registration

Our observations showed that some GP characteristics were associated with more frequent registration. First of all, it appears that male GPs register new patients at a higher rate, for both office care and house calls. Although this association has not previously been reported in the literature, it corresponds to our hypotheses, since the male GPs worked more hours and had more consultations weekly (thus offering a greater supply of care) than women [31]. Consequently, they should be registering new patients at a higher rate. It is nonetheless important to note that this association may partly reflect an age effect and not solely one of gender. That is, the youngest GPs express a desire to reduce their working time [32, 33], regardless of their gender [34]. This new generation of GPs has a higher proportion of women than any preceding generation [35]. It is thus possible that the gender effect we found is in part due to the effect of age on both male and female GPs.

We also showed that GPs practising alone agreed to register new patients more frequently than group practitioners, for both office and home care. Our result contrasts with the literature, specifically with a US study published in 2015 showing that access to a consultation with a GP for a patient new to the office was more frequent if the office housed either a group practice or patient-centred medical home (PCMH) [36]. On the other hand, it is consistent with our hypothesis about the care supply, since we know that GPs in solo practices have a higher number of consultations weekly (and, therefore, supply more care) than GPs in group practices [17]. In terms of interpretation, it is possible that GPs in a group practice find it easier to refuse to register new patients, because they can probably rely on potential associates who would agree to register new patients. This hypothesis requires corroboration by qualitative interviews.

#### Characteristics describing the practice neighbourhoods associated with registration

Some of our results concerning the neighbourhood characteristics can be compared with the literature. A study similar to ours, conducted in Canada, did not show that access to a consultation with a GP for a new patient was associated with the neighbourhood’s socioeconomic characteristics (ecological variable describing the inhabitants’ mean income). Nonetheless, this study had weak power, as it included only 375 GPs [21]. In studies where patients’ socioeconomic characteristics were studied individually (rather than ecologically, as we did), an association with deprivation at the bottom of the social hierarchy for access to consultations has been shown several times and supports our results. That is, the type of health insurance [19, 37], the type of jobs [21], and even the ethnic origin [38] reported by patients during telephone interviews are associated with lower access to office consultations among those at the bottom of the social hierarchy. Our work thus provides a supplementary perspective: this social discrimination is also present in the area and on a geographic and not solely an individual scale (the socioeconomic characteristics of patients were not specified in the scenario).

The area’s primary care supply characteristics, such as the number of consultations available annually or the distance to the nearest emergency department were not associated with registration. In our opinion this may reflect a phenomenon of saturation of GPs’ working time. That is, even in the tertiles, where the supply is greatest, GPs do not want to increase their workload and refuse to register the same proportion of new patients. This observation corroborates what they say when asked for their opinion about the GP supply near them: regardless of the supply, nearly half consider it inadequate [39].

On the other hand, the level of demand (unlike the supply) does appear to be associated with agreement to register new patients. That is, the areas where the population characteristics are likely to entail strong need for primary care (areas with greater deprivation and more patients with costly chronic diseases, and, therefore, high demand) are associated with a lower rate of agreement to register new patients. In these areas, GPs probably have to cope with higher demand. Solicited more often, those who do not want to increase their workload must probably refuse new patients more often.

The different directions of the association between population density and registration according to whether it is for office or home care raises questions. As GP density is higher in the areas with the highest population density, supply is greater there, and GPs should be led to refuse new patients less often. The more frequent refusal for registrations for house calls in the densest areas is probably linked to the fact that GPs in the most rural areas also perform the greatest number of house calls, as shown in France [40], the USA, and Germany [41].

Again for registration for house calls, the low number of characteristics associated with registration reflects, we think, the abandonment of this task by GPs, as seen in many Western countries since the second half of the twentieth century [18, 42, 43], in part because it takes up too much time (related to the phenomenon of saturation) and also because it is insufficiently remunerated [44, 45].

### Strengths and limitations

It is conceivable that the scenario’s predefined characteristics influenced the physicians’ decision about registration for home visits. That is, primary care doctors willing to register a new patient for office visits may be more inclined to accept a home registration at the same time, compared with a situation, where the request concerns only registration for home visits. For ethical and legal reasons, our model does not incorporate the data describing the characteristics of the work performed by the GPs we contacted. Among these, we did not access two important characteristics associated with their working time and, therefore, probably with the phenomenon that we are studying [46]: the size of their patient panel [46–48] and the social and demographic characteristics of their patients. For the same reasons, we did not have access to the GPs’ personal characteristics, in particular, their age. Nonetheless, we can hypothesise its associations with our outcomes with nonetheless several uncertainties. It is possible that GPs nearing retirement, seeking to reduce the size of their patient list, refuse new patients more often than their younger colleagues. There is, however, a competing hypothesis. Young GP work fewer hours than their elders [16] and are likely to obtain a patient panel of the size they want rapidly [49]. Moreover, younger physicians, concerned about maintaining their quality of life, keep themselves more distant from their patients’ demands than their elders and may be stricter in applying the rule to not accept new patients. To compensate for the lack of access to these data, we nonetheless used ecological variables for the municipality, specifically, the proportion of GPs aged 65 years or older; it was not associated with registration. To study the influence of these variables in the future, either qualitative surveys must be performed or another method used to obtain these data.

On the other hand, our work has an important strength: it is an exhaustive census of GPs practising in the region we are studying. The consideration of the levels of data in our analyses also allowed us to model as well as possible the phenomenon that we sought to analyse.

### Implications

Nearly half of all GPs reported that they were not accepting new patients for office care. This finding is especially disquieting given that the lack of registration with a GP is associated with a substantial reduction in patients’ access to both primary and secondary care and in the frequency of performance of prevention procedures [50, 51]. Given the possible consequences for the populations concerned, it appears essential to strengthen mechanisms to facilitate registration with GPs.

In our work, access to registration was not associated with the GP’s accessibility as estimated with gravity model [27, 28], which is nonetheless considered the most effective method of estimating access to primary care [27]. This indicator is also used in France to identify the areas where the supply of GPs is lowest and where the public authorities use economic incentives to try to encourage doctors to move into or not leave areas with doctor shortages [52]. The substantial fall in the numbers of GPs, since the turn of the century has confronted France with a penury of these primary care physicians [53], reported by both the population and the doctors [9]. Our work opens a pathway to improve the selection of areas to support. Because GPs refuse to register new patients more frequently in the most socioeconomically deprived areas, where the rates of both new arrivals and of the population with costly chronic diseases are highest, these three indicators should be integrated into the gravity model. This would make it possible to add a qualitative dimension to the population present and thus limit the risk of reinforcing social inequalities that can be engendered by reduced access to the health-care system for the populations of the most deprived neighbourhoods.

Our results concerning group practices seem to contradict the current policies that incite GPs to work together [36] in structures, where costs and health resource use are reduced [54, 55] and the health care supply supposedly higher [56, 57]. The grouping of GPs does not appear to be helpful to patients seeking a GP with whom to register. At a time when this type of organisation is needed to respond to the difficulties of access to primary care [58, 59], it will be interesting to follow the future changes in the rates of acceptance of these facilities promoted by the public authorities—and which appear to be growing.

### Research perspectives

It would have been interesting to conduct qualitative interviews of GPs. That is, while several variables were significant in our models, they explained less than 5% of the inter-GP variance (results not shown). This may be explained by the absence of important characteristics (GPs’ age and the time they spend working), but qualitative interviews could help to provide new clues.

This study focuses on the Île-de-France (Paris metropolitan) region, where the density of GPs is more than 11% below the national mean [60]. Although half the municipalities in this region are categorised as rural [61], only 5% of the region’s inhabitants live in these areas, which limits the representativity of the rural areas in our analysis.

Accordingly, the results must be interpreted with prudence and cannot be generalised across rural France. It thus appears necessary to expand the analyses of rural areas to grasp more accurately the disparities in access to care at the national scale. An ancillary study that we conducted in the district of Côtes-d’Armor (on the west coast of France) revealed that 61.2% of general practitioners were refusing to accept new patients.

Finally, finding a GP, especially for professional care at home, appears to be a prerequisite not always easy to meet and that can lead to limiting access to care or even to renouncing it. The fact that it is harder to find a GP in the most deprived areas with the most people with chronic diseases suggests the need to develop policies facilitating the settlement in these areas of new doctors.

Since 2023, the state has taken several actions to reduce the proportion of the population not registered with a GP (and thus having limited access to the health-care system). One of these enables the national health insurance fund (CNAM) to contact these patients directly to offer to put them into contact with GPs. State-subsidised ambulatory care organizations, including territorial professional health communities (CPTS), are also mobilised, sometimes with the direct involvement of GPs. Other countries, such as Canada, have set up a solution involving a one-stop shop that allows unregistered patients to be placed on a waiting list (with publicly criticised wait times) and be given a number to contact for an immediate referral when necessary**.** This study shows the need to enhance such mechanisms in the most disadvantaged and least densely populated areas**.** Moreover, since 2012, some physician remuneration (4–5% of their income in 2024) depends on the number of registered patients [62], as an incentive for GPs to increase the number of patients on their lists.

## Supplementary Information

Additional file 1.

## Data Availability

No data sets were generated or analysed during the current study.
