# Supplementary material for: Mismatch between registration possibilities and patients’ local health needs, a simulated patient survey in the Paris metropolitan area
Source: Hum Resour Health. 2025 Nov 3;23:59. doi: 10.1186/s12960-025-01020-4 (PMC12581403; doi:10.1186/s12960-025-01020-4)
Supplement: Supplementary file 1 — Additional file 1. [file 12960_2025_1020_MOESM1_ESM.docx]

**Predefined scenario**

- Interviewer: Hi, I'm calling to inquire, I just moved to the area and am looking for a new primary care physician. Do you accept new patients?

- Respondent: yes/no

* IF NO: end of conversation, thank you, goodbye.

* IF YES

- Interviewer: Okay, and secondly I wanted to know if you would be willing to follow my grandmother on a home visit as well?

- Respondent: yes/no

* IF NO: Me: Okay, thank you. Have a nice day.

* IF YES:

- Interviewer: okay, I will come back to you when I need. Thank you, have a nice day.
